# Linking Trait Differences to Community Dynamics: Evidence from *Eupatorium adenophorum* and Co-Occurring Native Species during a Three-Year Succession

**DOI:** 10.1371/journal.pone.0050247

**Published:** 2013-01-31

**Authors:** Xianming Gao, Yujie Zhao, Xuejun Yang, Shucun Sun

**Affiliations:** 1 State Key Laboratory of Vegetation and Environmental Change, Institute of Botany, Chinese Academy of Sciences, Beijing, China; 2 Graduate University of the Chinese Academy of Sciences, Beijing, China; 3 ECORES Lab, Chengdu Institute of Biology, Chinese Academy of Sciences, Chengdu, Sichuan, China; University of Nottingham, United Kingdom

## Abstract

Trait differences between invasive and native species are believed to be closely related to whether the former are successful. However, few studies have measured trait differences between invasive and native species directly under field conditions or during long term experiments. We examined the phenological pattern, plant height and biomass accumulation and allocation of Crofton weed (*Eupatorium adenophorum* Spreng.) and co-occurring native species in a community during a three-year succession. The phenological pattern of Crofton weed differed from that of co-occurring native species. Crofton weed had longer vegetative stage (when resources were more available), a higher biomass accumulation and a higher above/below-ground ratio compared to native species. Crofton weed was shorter than grasses and two forbs (*Artemisia tangutica* and *Cynoglossum amabil*e) during its first year of growth, but was significantly taller than all other species during subsequent years. The dominance (calculated as the importance value) of Crofton weed was the highest among all other species and continually increased over time while the dominance of co-occurring native species decreased. This study provides direct field evidence that trait differences are important to plant invasion.

## Introduction

As a result of increasing human activities, many species have invaded a wide range of new regions at an unprecedented rate [1]–[3]. The spread of invasive species and the homogenization of flora have been recognized as a global issue [4]–[8]. Exotic plants that become invasive can alter ecosystem structure and function [9]–[11], because invasive species may: (1) acquire resources differently from native species, (2) alter trophic relationships, or (3) change the frequency or intensity of disturbance [12]. Invasive species can also affect ecosystem processes through changes in plant-soil organism feedbacks [13], [14]. Thus, plant invasions pose major threats to biodiversity, ecosystem stability and human welfare [15], [16]. Consequently, mechanisms underlying invasiveness have become an important topic in ecology and conservation biology [9], [17]. Despite increasing efforts, it remains difficult to identify the mechanisms or even the functional traits that convey the ability of species to invade novel habitats [14], [16], [18]–[19].

It is clear nevertheless that functional traits (life form, phenology, polyploidy level, etc.) determine how plants reproduce and capture resources [20]–[22], which undoubtedly influence whether a species is successful when competing with other species for space and resources [9], [11], [17]. Because co-occurring invasive and native species experience similar environmental selection pressures (i.e. habitat filtering), several theories predict that they are likely to share traits adaptive to their local environment [17], [23]–[25]. At the same time, the success of invasive species may be due to trait differences [11], [23], [26]. In general, traits that enable high reproduction rates and rapid dispersal facilitate colonization while those that enable rapid growth and resource acquisition are important for establishment and the subsequent displacement of the resident vegetation [20], [27]. Functional traits related to physiology, biomass allocation, and size may also be related to invasiveness [28], [29].

The most successful invaders are often species with high specific leaf area (SLA), high phenotypic plasticity, high relative growth rate (RGR) and high nutrient turnover rate [3], [19], [30]–[31]. Thus, traits associated with growth and allocation can, in general, be used to predict interactions between introduced and native species in a particular environment [32], [33]. A recent study suggests that many plant attributes play an important role in determining the competitive interactions between native and invasive exotic plants, and that an understanding of these traits may be informative for predicting the outcome of interactions between species [32]. Because displacement of native species by invasive species has the greatest potential to impact community structure and function, we need a better understanding of whether the traits relating to establishment are correlated with an increased abundance of invading species [17].

Invasion biologists and community ecologists have long recognized the role of phenology (the timing of periodic life-history events) in promoting the establishment and spread of exotic species [34]–[36]. The phenology of a plant species is closely related to many characteristics that are important to understanding plant invasion. For example, leaf budburst and senescence strongly correlate with not only with when species acquire light and soil resources but also when they are at the greatest risk of herbivory [37], [38]. In addition, the period of flowering determines when and if invasive and native species compete for generalist pollinators [39]. Therefore, phenology is an important factor controlling community assembly and patterns of species invasion [40].

In order to examine whether trait differences play important roles in determining whether a species is a successful invader, we elected to study Crofton weed [*Eupatorium adenophorum* Spreng.  =  *Ageratina adenophora* (Spreng.) King and H.E. Robins.], which is a perennial semi-shrub native to Mexico and Costa Rica that has invaded more than 30 countries and regions in the tropical and subtropical zones of the world, such as America, Australia, New Zealand, southern Africa, India and China [41]–[44]. The species can create monospecific stands and is considered a serious threat to biodiversity and ecosystem function [41], [43]–[46]. In the field, Crofton weed is much taller and more abundant than most of the co-occurring species in invaded communities (Xianming Gao, Personal observation). Thus, we hypothesized that differences in plant height as well as biomass accumulation and phenology may play an important role in the success of Croften weed. Based on this speculation we asked three main questions: (i) Do phenological events (leafing, flowering, and senescing) differ between Crofton weed and native species? (ii) Is Crofton weed taller and more adroit at biomass accumulation than co-occurring native species? and (iii) Do trait differences correlate with Crofton weed invasive success?

## Materials and Methods

### Ethics Statement

All necessary permits were obtained from the Panzhihua Forestry Bureau of Sichuan Province, which has responsible for our field study site.

### Study site

This study was conducted in an area of grazed shrubland in Renhe District of Panzhihua (26°10′–26°11′ N, 101°47′–101°48′ E; 1 500–3 000 m asl), south of Sichuan Province, China. This area is characterized by a semi-arid monsoon climate of south subtropical zone with pronounced wet and dry seasons. This area experiences a rainy season from June to October, and there is significantly less rain during the rest of the year. Mean annual precipitation ranges between 760 and 1 100 mm, and mean annual temperature is 20.4°C, with mean monthly temperature ranging from 11°C in December to 27°C in July. Mean annual sunshine time is 2 745 h and frost-free period 300 d. Crofton weed has invaded 10.92% of the total land area in Panzhihua in 2002 [44].

Crofton weed is a perennial semi-shrub in the family of Asteraceae. It was first introduced to Yunnan Province in China from Burma in the 1940s and became widespread in Southwest China after several decades [44], [46]. A shrub community previously dominated by Crofton weed with homogeneous vegetation was selected to conduct the field study. This community consisted of Crofton weed and fourteen co-occurring native species out of which eleven are deciduous and four are evergreen. Of the fourteen native species, *Sida szechuensis*, *Rumex hastatus*, *Myrsine africana*, *Pistacia weinmannifolia*, *Coriaria nepalensis*, *Jasminum subhumile* and *Dodonaea viscose* are shrubs, *Cynoglossum amabile*, *Geranium pylzowianum*, *Artemisia tangutica*, *Carpesium divaricatum* and *Pimpinella diversifolia* are forbs, and *Digitaria sanguinalis* and *Setaria viridis* are grasses (Table 1). Among the native species, *Artemisia tangutica* and *Carpesium divaricatum* were the members of the same family (Asteraceae) as the alien invasive Crofton weed (Table 1). The distribution ranges of the native species were either greater than or overlapped that of Crofton weed in China.

**Table 1:** List of species with abbreviations in the study site.

Abbrev.	Species	Family	Life form
Ea	*Eupatorium adenophorum* Spreng.	Asteraceae	semi-shrub
Ds	*Digitaria sanguinalis* (L.) Scop.	Gramineae	grass
Sv	*Setaria viridis* (L.) Beauv.	Gramineae	grass
At	*Artemisia tangutica* Pamp.	Asteraceae	forb
Cd	*Carpesium divaricatum* Sieb. et Zucc.	Asteraceae	forb
Ca	*Cynoglossum amabil*e Stapf et Drumm.	Boraginaceae	forb
Gp	*Geranium pylzowianum* Maxim.	Geraniaceae	forb
Pd	*Pimpinella diversifolia* DC.	Umbelliferae	forb
Ss	*Sida szechuensis* Matsuda	Malvaceae	semi-shrub
Rh	*Rumex hastatus* D. Don	Polygonaceae	shrub
Ma	*Myrsine africana* L.	Myrsinaceae	shrub
Pw	*Pistacia weinmannifoli*a J. Poisson ex Franch.	Anacardiaceae	shrub
Cn	*Coriaria nepalensis* Wall.	Coriariaceae	shrub
Js	*Jasminum subhumile* W. W. Smith	Oleaceae	shrub
Dv	*Dodonaea viscosa* (L.) Jacq. Enum	Sapindaceae	shrub

### Experimental design

This study ran for three years. In October, 2004, we enclosed 1.5 ha of flat (0–5°) shrub and fenced the land to prevent grazing and human disturbance. The fenced land was then divided into: (1) 0.3 ha (60 m ×50 m) reserved for phenological observation; (2) 1.2 ha (60 m ×200 m) for invasion succession experiment.

We removed all plants manually in the 1.2 ha area selected for the simulation experiment of invasion succession. This area was divided into four 50 m ×60 m blocks each of which contained four 10 m ×50 m plots with a 5 m buffer from each plot edge. Five 5 m ×5 m quadrats were placed in each plot. In each block, one of the four plots were used to nondestructively monitor abundance, coverage and frequency of each species (nondestructive plots) each November each year; the other three plots were harvested destructively to determine plant biomass and soil throughout the three-year-long experiment (destructive plots). Abundance was calculated as the number of individuals in each of the 5 quadrats; coverage was calculated as the percentage of the are projected in the vertical plane in each quadrat. A quadrat was further divided into twenty five 1 m ×1 m temporary grids and plant frequency was measured as the number of grid cells occupied by each species. Relative abundance was calculated as the abundance of a species divided by sum of the abundance of all species in a quadrat. Relative coverage and relative frequency were calculated similarly. Thus, each nondestructive plot had 5 measures of relative abundance, relative coverage and relative frequency, which were averaged before analysis. All new individuals came from the soil seed bank. Destroyed plots were marked and not subsequently sampled.

### Measurements of soil conditions and plant traits

We randomly collected three soil samples at a depth of 0–20 cm from each destructive plot in each block, using core sampler measuring 50.46 mm in diameter and 50 mm in length. Soil samples from the same plot were mixed to obtain a representative overall sample, which were subsequently air-dried, pulverised, sieved through 0.15 mm screens and stored at room temperature for soil nutrient analyses. Soil pH (1: 1 w/v water) was determined with a glass electrode. Soil total N was measured using the Kjeldahl method. Soil total P was determined using the colorimetric method as described by Murphy and Riley (1962). And soil total K was measured using the flame photometry method [47]. Soil available NO_3_^-^N, available NH_4_^-^N, available P and available K were analyzed by means of the phenol disulfonic acid method, the indophenol blue colorimetric method, Olsen-P method and the NH_4_OAc method, respectively [48].

Plant height and biomass allocation patterns were measured once every year. In each destructive plot of each block, 30 newly emerged seedlings per species were randomly tagged in November, 2004. In November of 2005, 2006, and 2007, plant height of tagged individuals of each species per plot were measured. Belowground biomass was measured by excavating fifteen 25 cm ×25 cm soil cores at a depth of 30 cm. All roots were washed and sieved, and separated for both Crofton weed and native species. The dry mass of above- and belowground organs was determined after oven-drying for 48 h at 70°C. Biomass allocation was calculated as the ratio of below-ground biomass to above-ground biomass.

The phenology of Crofton weed and co-occurring species was measured in the 0.3 ha reserved area. Twenty mature individuals per species were randomly selected and examined to determine growth initiation (based on the sprouting of buds and the germination of seeds), the duration of the vegetative phase, and the timing of flowering, fruiting and senescence. Detailed records for each of these “phenophases” were kept for all of the study species based on data collected at 10- to 14-day intervals between November, 2004 and November, 2007. Although a phenophase was considered to have started if it was observed in at least 10% of all of the individuals representing each species, the phenology of the individuals of each species was strongly synchronized and distinctive for each of our study species.

### Data analysis

Soil pH, total nutrients and available nutrients across the four blocks were analyzed by ANOVA to evaluate the homogeneity of soil conditions in the experimental site. The length of the growing season significantly varied among different species, but growth rates measured as accumulated plant height and biomass over the same period of time did not, i.e., growth rates were comparable among all species. To assess the overall significance of a species in vegetation dynamics, we calculated importance value (IV) as: IV  =  [(relative abundance + relative frequency + relative coverage)/3] ×100 [48]. Differences in height, biomass and biomass allocation across species were analyzed with two-way ANOVA, in which “species” was a fixed factor and “year” was a random factor. Because IV was measured in the same nondestructive plot over three years, the species IV data were evaluated by repeated measure ANOVA using the general linear model (GLM) procedure. Sphericity was checked using the Mauchly test. Since Mauchly's test was significant (*P*<0.001, df  = 2), all within-subject results incorporated the Greenhouse-Geisser correction. For multiple comparisons, Tukey's HSD test was used. Data were log-transformed for plant height and biomass when they would improve normality. All analyses were conducted with SPSS 17.0 for Windows (SPSS Inc., Chicago, IL).

## Results

### Soil conditions

Soil pH (6.32±0.34), total N (1.56±0.25 g·kg^−1^), total P (0.82±0.13 g·kg^−1^), total K (9.05±0.48 g·kg^−1^), NO_3_^−^N (28.53±0.22 mg·kg^−1^), NH_4_^−^N (17.06±3.43 mg·kg^−1^), available P (7.26±0.55 mg·kg^−1^) and available K (50.44±4.58 mg·kg^−1^) did not differ among the four blocks (Table 2), indicating a homogeneous soil environment in the study site.

**Table 2:** ANOVAs for soil homogeneity in the study site.

Variable	Source	df	MS	*F*	*P*
pH	between groups	3	0.15	1.44	0.28
	within groups	12	0.10		
total N	between groups	3	0.13	2.80	0.09
	within groups	12	0.05		
total P	between groups	3	0.01	0.41	0.75
	within groups	12	0.02		
total K	between groups	3	0.03	0.12	0.95
	within groups	12	0.28		
NO_3_^−^N	between groups	3	0.09	0.16	0.93
	within groups	12	0.59		
NH_4_^−^N	between groups	3	1.99	0.39	0.77
	within groups	12	5.17		
available P	between groups	3	0.03	0.07	0.97
	within groups	12	0.37		
available K	between groups	3	0.17	0.01	0.10
	within groups	12	15.29		

### Phenological spectra

**Figure 1:**
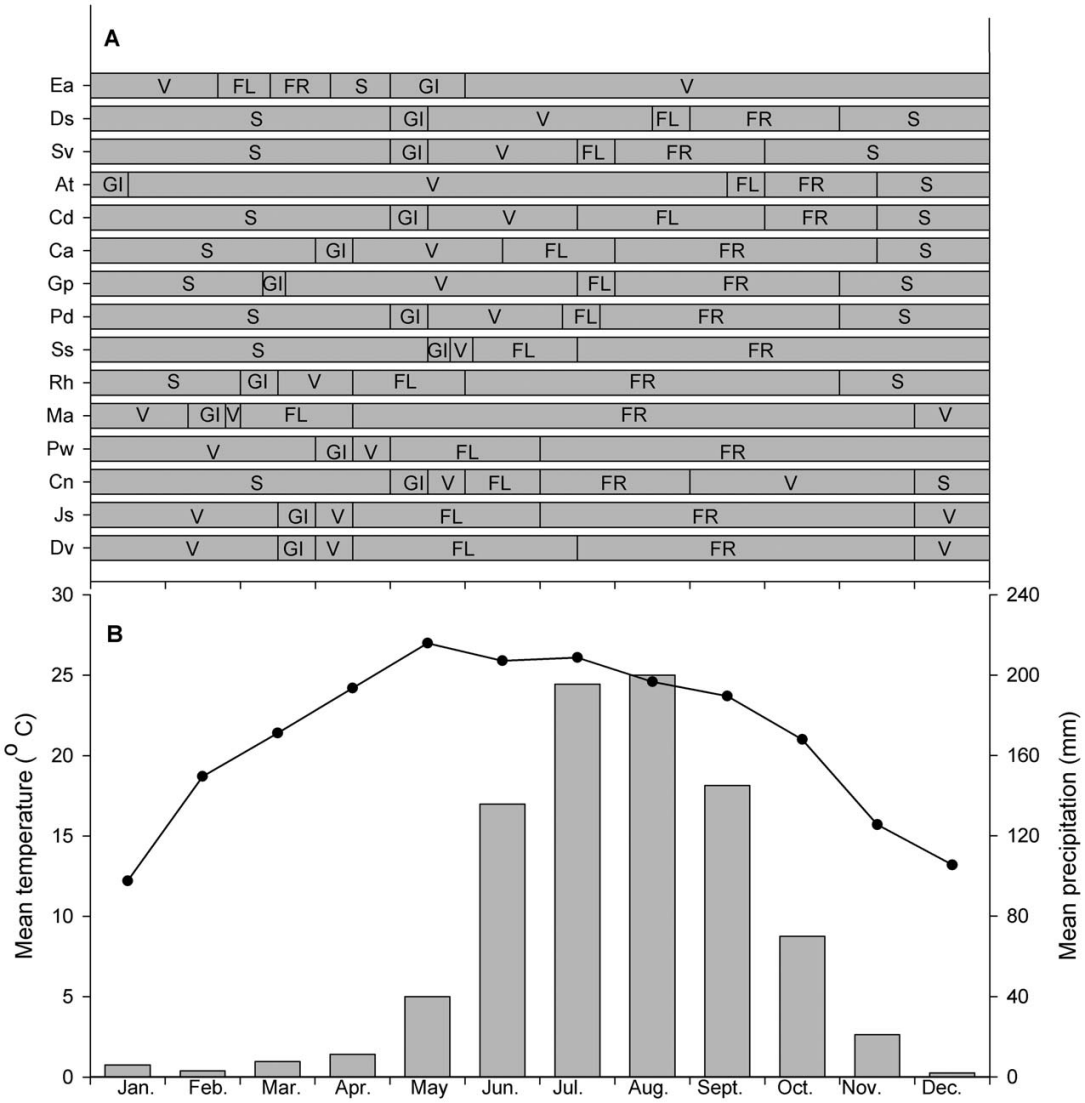
Phenophases of the co-occurring species and climatic information of the study site. Panel A: phenological spectrums of Crofton weed and 14 co-occurring native species in the study site. Panel B: mean precipitation (bars) and temperature (curves) data for the study site from 2004 to 2007. Species abbreviations are given in Table 1. GI  =  Growth initiation; V  =  Vegetative; FL  =  Flowering; FR  =  Fruiting; S  =  Senescence.

Of the fifteen species studied (Table 1), contrasting phenological spectra were observed between Crofton weed and the co-occurring native species (Fig. 1a). In four deciduous species (*A. tangutica*, *C. amabile*, *G. pylzowianum* and *R. hastatus*) and four evergreen species (*S. szechuensis*, *M. Africana*, *D. viscosa* and *P. weinmannifolia*), growth was initiated before mid-April and growth cycles (from the beginning of growth to the end of fruiting) ranged between 7.5 to 11.5 months (Fig. 1a). Growth initiation for the other seven deciduous species, including Crofton weed, occurred in early May when the daily temperature began to rise (Fig. 1). Growth cycles of the seven species were completed within a 7-month period before the onset of the dry season in January except that of Crofton weed, which extended to 11 months.

For Crofton weed and six native species, flowering began and was completed before the onset of the rainy season in June (Fig. 1). All native species set seed during the rainy season between June and October, with the exception of Crofton weed, which set seed during the dry season (Fig. 1). The reproductive growth phases (from the initiation of flowering to the end of fruiting) of all native species were more than two months, with 50% more than four months and 43% more than six months. In contrast, the reproductive growth phase of Crofton weed was less than two months (Fig. 1a).

### Plant height during three successive years

**Figure 2:**
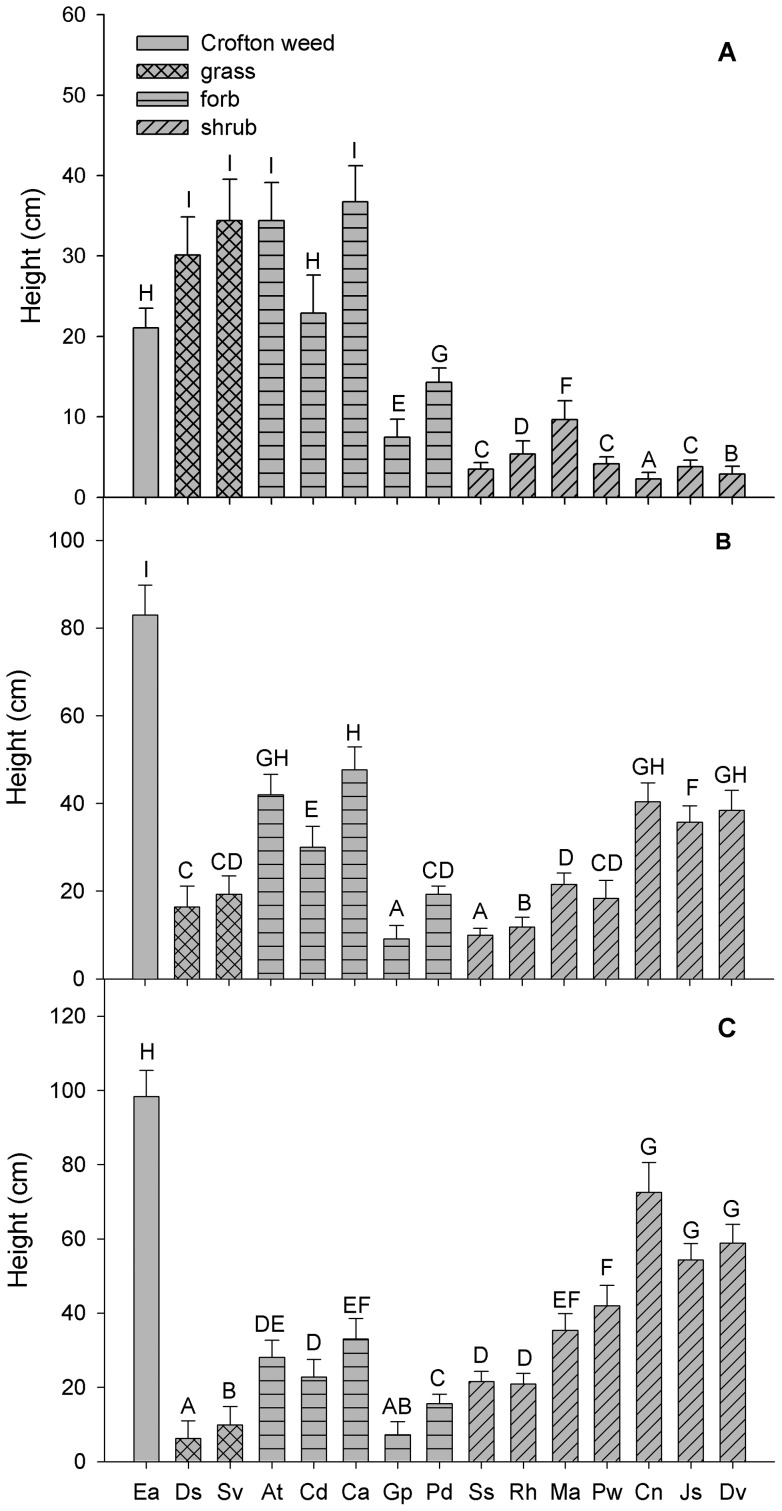
Plant height for Crofton weed and the co-occurring native species in the study site. Panel A, B, and C represent plant height for Crofton weed and the co-occurring native species in the first, second, and third year, respectively. Species abbreviations are given in Table 1. Different letters above bars indicate significant differences (*P*<0.05). Error bars denote 1 standard error.

We found significant differences in plant height among the fifteen species in each of the three successive years (Fig. 2, Table 3). In the first year, the height of Crofton weed was 21.07±2.45 cm, while the mean height values for other species ranged from 2.27±0.81 cm to 36.73±4.49 cm (Fig. 2a). Crofton weed was significantly taller than the native shrubs, but was shorter than grasses and two forbs (*A. tangutica* and *C. amabil*e) in the first year of growth (Fig. 2a). In the second year, the height of Crofton weed was 82.93±6.86 cm, which exceeded the height of all of the other species examined (Fig. 2b). Finally, Crofton weed was significantly taller (98.4±7.03 cm) than any of the other native species during the third year (Fig. 2c). Specifically, Crofton weed was about 25 cm taller than *C. nepalensis*, 40 cm taller than *J. subhumile* and *D. viscose*, 60 cm taller than *C. amabile*, *M. africana* and *P. weinmannifolia*, and more than 70 cm taller than the other native species.

**Table 3:** Two-way ANOVAs for plant height, biomass and biomass allocation (below/above–ground biomass) during three successive years in study sites.

Variable	Source	df	MS	F	P
Height	Species	14	29.89	2.21	0.03
	Year	2	591.45	43.64	<0.001
	Species × year	28	13.55	36.96	<0.001
Biomass	Species	14	6.17	5.76	<0.001
	Year	2	74.04	69.06	<0.001
	Species × year	28	1.07	31.46	<0.001
Biomass allocation	Species	14	3.42	16.36	<0.001
	Year	2	0.20	0.94	0.41
	Species × year	28	0.21	27.84	<0.001

The overall increment in plant height of Crofton weed was far greater than that of the native species during each of the successive years (Fig. 2). The height of Crofton weed and the seven native shrubs increased significantly during the three years (Table 3). However, the height of *D. sanguinalis* and *S. viridis* decreased significantly in the second year and that of *A. tangutica*, *C. divaricatum*, *D. sanguinalis*, *S. viridis*, *C. amabile* and *P. diversifolia* in the third year (Fig. 2; Table 3).

### Plant biomass during three successive years

**Figure 3:**
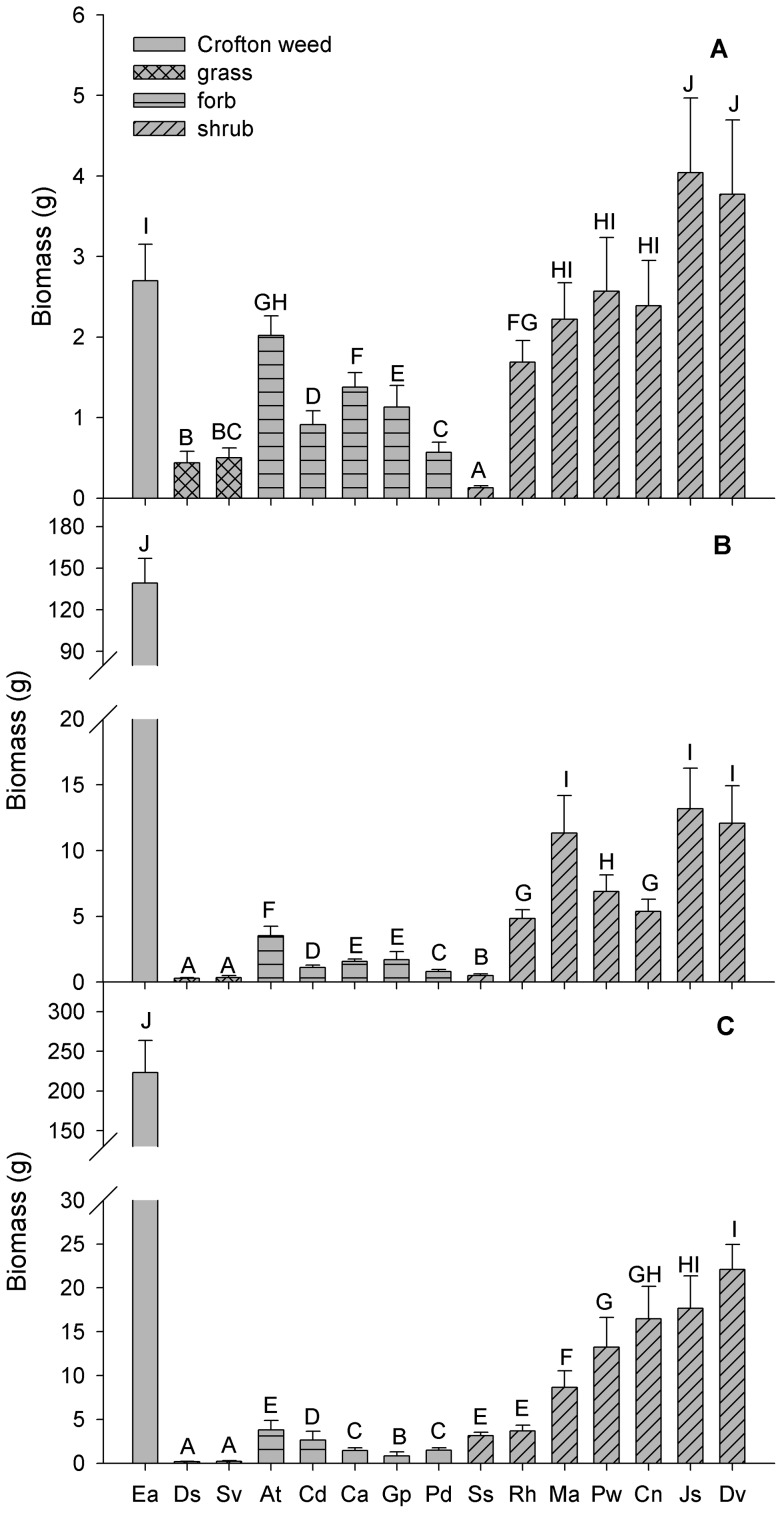
Plant biomass for Crofton weed and the co-occurring native species in the study site. Panel A, B, and C represent plant biomass for Crofton weed and the co-occurring native species in the first, second, and third year, respectively. Species abbreviations are given in Table 1. Different letters above bars indicate significant differences (*P*<0.05). Error bars denote 1 standard error.

Plant biomass differed significantly among the 15 species; Crofton weed had significantly more biomass than that of any of the native species with the exception of *M. africana*, *P. weinmannifolia*, *C. nepalensis*, *J. subhumile* and *D. viscose* in the first year (Fig. 3; Table 3). In the first year, the biomass of Crofton weed was greater than that of 9 native species including all of the herbs and two shrubs (*S. szechuensis* and *R. hastatus*) but it was smaller than that of the two grasses (Fig. 3a). In the second year, the biomass of Crofton weed (139.17±18.00 g) was 10 times greater than that of *J. subhumile* (which had the largest biomass among the native species) (Fig. 3b). In the third year, the biomass of Crofton weed was about 9 times greater than that of *D. viscose* (which had the largest biomass among the native species; Fig. 3c).

Both the overall and the annual increment in the biomass of Crofton weed exceeded that of the native species during the three successive years with the exception of the annual increment of *J. subhumile* and *D. viscose* in the first year (Fig. 3). Moreover, the biomass accumulation of *D. sanguinalis* and S. *viridis* decreased significantly in the second year as it did for *D. sanguinalis* and *S. viridis*, *G. pylzowianum*, *R. hastatus* and *M. africana* during the third year (Fig. 3; Table 3).

### Biomass allocation

**Figure 4:**
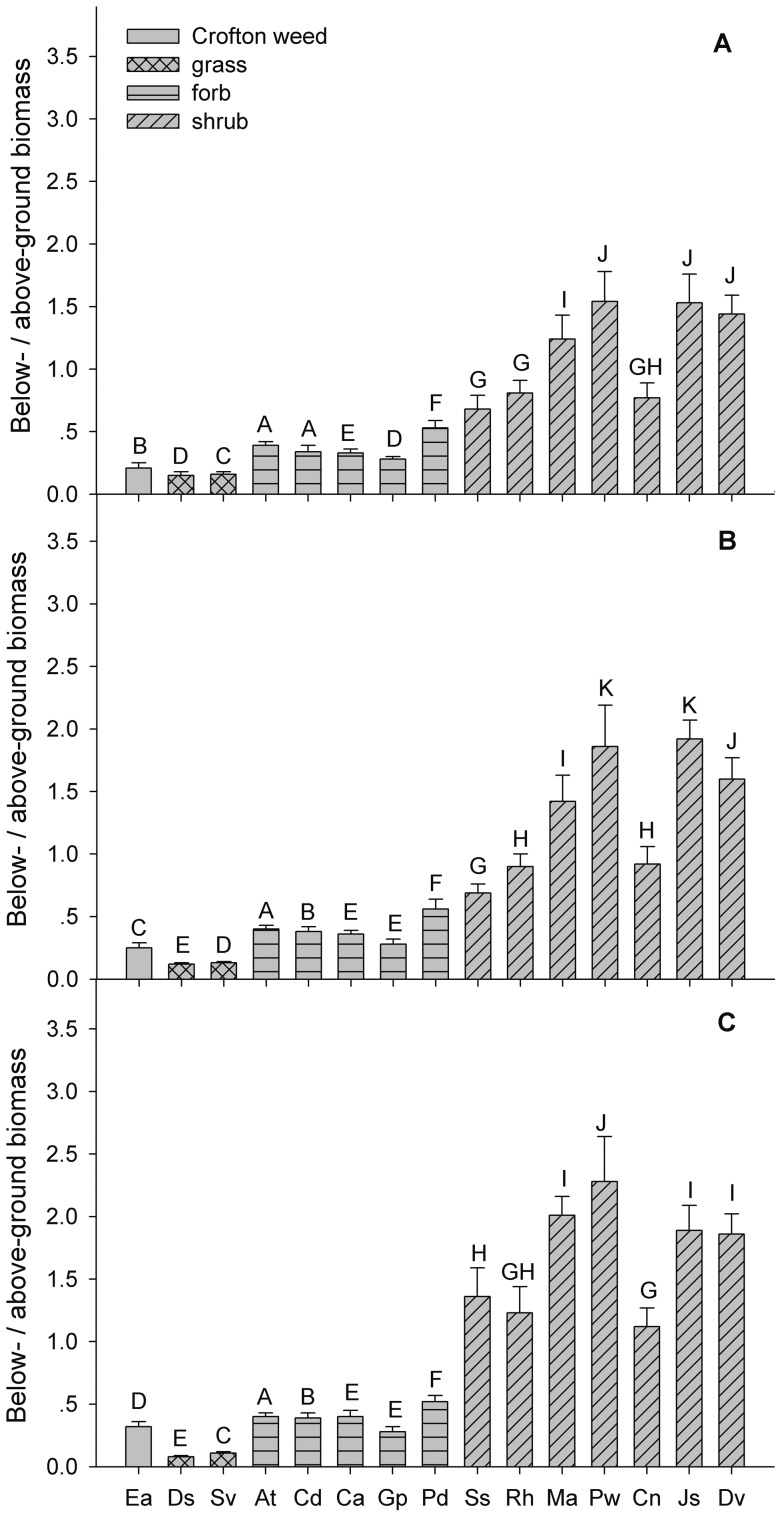
Biomass allocation for Crofton weed and the co-occurring native species in the study site. Panel A, B, and C represent below/above-ground biomass for Crofton weed and the co-occurring native species in the first, second, and third year, respectively. Species abbreviations are given in Table 1. Different letters above bars indicate significant differences (*P*<0.05). Error bars denote 1 standard error.

The ratio of below- to aboveground biomass differed significantly among the species and did not obviously change across the three years (Fig. 4; Table 3), i.e., a relatively constant biomass allocation pattern was observed for each species. For most native shrubs such as *M. africana*, *P. weinmannifolia*, *J. subhumile* and *D. viscose*, more biomass was allocated to belowground organs than to aboveground organs. The reverse was true for Crofton weed and the native herbs. The biomass allocation of Crofton weed was similar to that of the native grasses but differed from that of the native shrubs, i.e., belowground biomass was less than one third that of aboveground biomass for Crofton weed and *D. sanguinalis* and *S. viridis*, while it was more than half that of the aboveground biomass of *S. szechuensis*, *R. hastatus*, *M. africana*, *P. weinmannifolia*, *C. nepalensis*, *D. sanguinalis* and *S. viridis* (Fig. 4). Moreover, the ratio of below- and aboveground biomass of Crofton weed was significantly lower than that of all of the native species with the exception of *D. sanguinalis* and *S. viridis* (Fig. 4). Thus, Crofton weed had a pronounced bias toward aboveground biomass allocation.

### Community succession in three years

**Figure 5:**
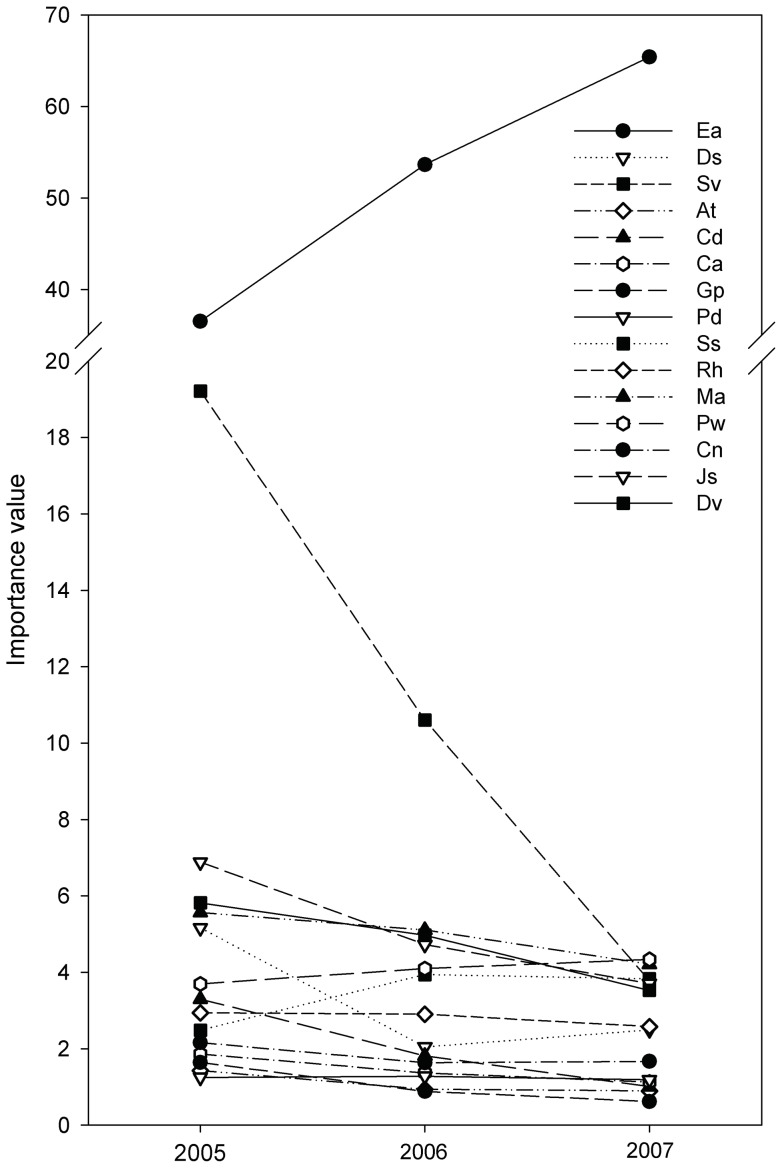
Inter-annual variations in importance value for Crofton weed and the co-occurring native species in the study site.

The importance value differed significantly among the fifteen species during each of the three successive years (Table 4). Crofton weed was dominant in the first year after disturbance and maintained a high importance value throughout the three years (Fig. 5; Table 4). Compared to the highest importance value among the native species (i.e., *S. viridis*), Crofton weed was 17.32 higher in the first year, 53.64 in the second year and 61.08 in the third year, respectively (Fig. 5).

**Table 4:** Repeated measure ANOVA for species importance value during three successive years in the study site.

Source		df	MS	*F*	*P*
**Between subjects**	Species	14	2.30	199.83	<0.001
	Error (species)	45	0.01		
**Within subjects**	Time	1.43	0.66	384.56	<0.001
	Time × species	20	0.11	61.74	<0.001
	Error (time)	64.44	0.00		

The Greenhouse-Geisser corrected degrees of freedom and F-statistic were reported for within-subjects comparison.

The analyses also revealed opposite population dynamics for native and the invasive Crofton weed during the community succession, in which importance value of Crofton weed kept increasing year by year while the co-occurring natives decreased. Both the overall and the annual increment in the importance value of Crofton weed was far greater than those of the co-occurring native species during each of the three years (Fig. 5; Table 5). Moreover, the significantly continuous increase in the importance value of Crofton weed was accompanied by a significant decrease in the importance value of each of the 9 co-occurring native species; no change was observed for *P. diversifolia*, *R. hastatus*, *P. weinmannifolia* and *S. szechuensis* (Table 5). Among the native species, the importance value significantly decreased year by year for all of the herbs with the exception of *A. tangutica* in the third year. The importance value also significantly decreased for *A. tangutica* and *C. nepalensis* in the second year and for *M. africana* and *D. viscose* in the third year (Table 5).

**Table 5:** Annual increment in importance value (IV) for species during three successive years in the study site.

Species	IV increment		
	**04–05**	**05–06**	**06–07**
Ea	**36.53**	**17.12**	**11.78**
At	**1.42**	**−0.48**	−0.05
Cd	**3.30**	**−1.49**	**−0.80**
Ds	**5.16**	**−3.12**	**0.45**
Sv	**19.21**	**−8.61**	**−6.83**
Ca	**1.86**	**−0.50**	**−0.24**
Gp	**1.64**	**−0.76**	**−0.26**
Pd	**1.25**	0.03	−0.09
Ss	**2.48**	**1.46**	−0.11
Rh	**2.94**	−0.04	−0.32
Ma	**5.56**	−0.46	**−0.90**
Pw	**3.70**	0.40	0.24
Cn	**2.16**	**−0.53**	0.03
Js	**6.88**	**−2.16**	**−1.02**
Dv	**5.82**	−0.85	**−1.44**

Species abbreviations are given in Table 2. Significant increments (*P*<0.05) are shown in bold.

## Discussion

Despite its importance, the number of studies comparing the functional traits of invasive and non-invasive species grown under common environmental conditions is still relatively low [3] and comparisons have seldom been made under field conditions or in long-term studies. Our study shows that Crofton weed becomes the dominant species after a mere three year period as a consequence of critical traits that differ significantly from those of native species. Thus, our study is one of a very few showing that trait differences between invasive and native species establish a route by which a species can invade a local ecosystem.

Phenology plays an important role in the success of exotic species [36], [40], and studies have shown that invasive species, when compared with native species, possess different phenological characteristics that confer advantages [35]. In our study, we found contrasting phenological patterns between Crofton weed and co-occurring native species. In particular, Crofton weed flowers earlier, maintains a longer vegetative growth phase and a shorter reproductive phase compared to its native counterparts (Fig. 1). Such “seasonal” priority effects have been suggested as an important mechanism facilitating the success of invasive plants by promoting rapid growth and the dissemination of seeds or fruits [39], [40], [49]–[51]. For example, earlier flowering would promote plant performance and reproductive output and result in a longer growing season and potentially higher productivity than later flowering [39], [51]. By the same token, differences in phenology can allow an introduced species to compete more successfully for light, soil nutrients, water and other resources in comparison to native species [52], [53]. In our study, the vegetative growth of Crofton weed occurs during the favorable rainy season, which fosters the accumulation of biomass. In contrast, native species entered into their reproductive phase (especially fruiting) at the same time which resulted in the consumption of larger proportions of energy and thus lower biomass accumulation compared to Crofton weed (Fig. 1). In addition, Crofton weed had a longer vegetative growth phase and a shorter reproductive growth phase than did the native species. Extending the former is beneficial to light capture and thus biomass accumulation [54], [55], whereas a shorter reproductive growth phase confers a competitive advantage over native species.

Increasing plant height can served as a strategy for light competition but it can also impose a cost in terms of structural support and water transport [31], [56]–[58]. Although the difference in height between Crofton weed and the other species was not significant during the first year, the longer vegetative stage of Crofton weed resulted in an steady and significant gain in plant height during each of the successive two years (Fig. 2), which resulted in superior light capture and the suppression of the growth of native species.

Crofton weed had a significantly greater biomass than most of the native species and its annual increment in biomass was far greater than most of the native species during each of the three successive years (Fig. 3). Thus, Crofton weed has a higher growth rate largely because of a close relationship between plant height and biomass accumulation and allocation, as suggested by other workers [33], [58]. In passing, it should be noted that successive observations of a three year period were required to observe this phenomenology, which would not have been obvious had the experiment proceeded for a single year.

The relationship between plant height and accumulated biomass is correlated with the biomass allocation patterns of all of the species studied here. It has been suggested that greater biomass accumulation is crucial for successful seedling establishment, while the outcome of competition between plant species depends more on biomass allocation patterns [33], [59]. In this context, the biomass allocation of Crofton weed was similar to that of the native grasses but differed from that of the native shrubs, i.e. Crofton weed allocate a larger proportion of its biomass to aboveground organs than to belowground organs (Fig. 4). This “strategy” has been reported previously in studies that have shown that invasive plants have much lower root-shoot ratios than native species do [14], [16], [60]. Indeed, a greater biomass accumulation in above- as opposed to belowground organs largely accounts for the greater height of Crofton weed compared to the native species in our study site.

The aforementioned helps understand why the importance value of Crofton weed steadily increased over the three year period while those of the co-occurring natives decreased (Fig. 5), which indicates that Crofton weed gradually excluded all of the natives. However, an important observation is that the coverage of Crofton weed was much larger than all of the co-occurring species during each successive year, but the abundance (i.e., number of individual plants) of the weed was not the greatest. In tandem, these observations indicate that Crofton weed grows to dominate due to the accumulation of biomass that results in bigger plants and not as a result of an increase in the number of plants. In turn, this suggests that seedling establishment of the weed is not particularly successful, but that, once a plant gets established, it outcompetes its neighbors rapidly (likely as a result of shading).

In conclusion, our three-year study shows that trait differences between Crofton weed and co-occurring native species contributes to the invasiveness of the weed. In particular, phenological differences might have allowed Crofton weed to accumulate more biomass and grow taller than co-occurring native species. Crofton weed allocates more biomass to its aboveground organs that in turn promotes a rapid growth in height resulting in a greater capacity to harvest light, which further promotes biomass accumulation at the level of individual plants. This “strategy” allows the weed to displace neighboring native plants. Our data also indicate that Crofton weed gains dominance not by increasing in population size (the number of individuals in a site), but rather by increasing the size of individual plants that become established after disturbance. Future work is required to determine whether these features can be generalized to other study sites.
